# Draft Genome Sequences of Nine Japanese Strains of the Kiwifruit Bacterial Canker Pathogen Pseudomonas syringae pv. actinidiae Biovar 3

**DOI:** 10.1128/MRA.01007-20

**Published:** 2020-11-05

**Authors:** Takashi Fujikawa, Hiroyuki Sawada

**Affiliations:** a Institute of Fruit Tree and Tea Science, National Agriculture and Food Research Organization, Tsukuba, Ibaraki, Japan; b Genetic Resources Center, National Agriculture and Food Research Organization, Tsukuba, Ibaraki, Japan; University of Arizona

## Abstract

Pseudomonas syringae pv. actinidiae is the pathogen that causes kiwifruit bacterial canker and is categorized into several groups (biovars). In Japan, biovar 3, known as the pandemic group, was first discovered in 2014. Here, we sequenced the genomes of nine Japanese biovar 3 strains.

## ANNOUNCEMENT

The kiwifruit bacterial canker pathogen, Pseudomonas syringae pv. actinidiae, causes serious damage to kiwifruit production worldwide (1) and is currently subdivided into several groups (biovars) (2, 3). Among them, biovar 3 caused recent pandemics of this disease in various parts of the world (1–5). In Japan, biovar 3 strains were first discovered in 2014 (6) and have caused enormous damage since then (3). Because biovar 3 strains were not detected at all in Japan until 2014 (3, 6) and it has been clarified that the pandemic lineage of biovar 3 originated in China (4, 5), it is speculated that biovar 3 might have invaded Japan from any country where it previously occurred (3). In biovar 3, various types of the integrative and conjugative element (ICE) with differing structures and insertion sites have been detected (5). Among them, Pac_ICE1 was detected by PCR assays in biovar 3 strains isolated in Japan (6). Pac_ICE1 has also been detected in biovar 3 strains isolated in China and New Zealand (5). Here, we selected nine Japanese strains of biovar 3 from the National Agriculture and Food Research Organization (NARO) Genebank collection (MAFF collection) (https://www.gene.affrc.go.jp/index_en.php), whose MAFF accession numbers are found in Table 1, and sequenced their genomes to help elucidate the origin, evolution, transmission, and pathogenicity of biovar 3.

**TABLE 1:** Genome data and accession numbers for nine strains of *Pseudomonas syringae* pv. actinidiae biovar 3

MAFF accession no. (strain)	Isolation host	Isolation area, yr	GenBank accession no.	Genome size (bp)	G+C content (mol%)	No. of contigs	*N*_50_ (bp)	Total no. of genes^*a*^	No. of rRNAs (5S, 16S, 23S)^*a*^	No. of tRNAs^*a*^	SRA^*b*^ accession no.	No. of reads	Avg read length (bp)	Genome coverage (×)
MAFF 212101	*Actinidia chinensis*	Saga Prefecture, 2014	PGSP00000000	6,112,610	58.6	461	24,702	5,931	4, 1, 1	38	SRR11744872	2,256,006	152.0	54.7
MAFF 212104	*A. chinensis*	Ehime Prefecture, 2014	PGSX00000000	6,135,799	58.6	489	23,513	6,021	2, 1, 1	46	SRR11744878	4,126,079	241.2	159.9
MAFF 212109	*A. chinensis*	Wakayama Prefecture, 2014	PGSS00000000	6,142,013	58.6	457	25,821	6,081	3, 1, 2	48	SRR11744873	6,152,457	280.9	278.1
MAFF 212111	*A. chinensis*	Fukuoka Prefecture, 2014	PGSQ00000000	6,134,312	58.5	428	29,979	6,019	3, 1, 1	40	SRR11744874	4,591,637	269.7	198.8
MAFF 212115	*A. chinensis*	Fukuoka Prefecture, 2014	PGSO00000000	5,786,754	58.7	495	22,601	5,662	2, 1, 1	37	SRR11744870	1,763,341	181.5	54.0
MAFF 212118	*A. chinensis*	Fukuoka Prefecture, 2014	PHQZ00000000	4,223,244	58.6	751	12,356	5,847	1, 1, 2	37	SRR11744871	1,002,668	172.0	52.5
MAFF 212145 (Saga2-1)	*A. chinensis*	Saga Prefecture, 2014	PGSV00000000	6,052,839	58.5	632	16,127	5,980	2, 1, 2	38	SRR11744876	3,753,601	172.2	38.8
MAFF 212357 (1404)	*Actinidia deliciosa*	Shizuoka Prefecture, 2014	PGSZ00000000	6,127,733	58.6	408	28,264	5,981	3, 2, 1	52	SRR11744880	3,200,751	187.1	96.4
MAFF 212440 (psa142027)	*A. chinensis*	Ehime Prefecture, 2014	PGSW00000000	6,130,793	58.5	481	23,234	6,018	4, 2, 1	54	SRR11744879	2,432,513	223.1	85.8

a As determined by NCBI PGAP annotation.

b SRA, Sequence Read Archive.

Genomic DNAs of the nine strains were prepared and sequenced following the methods of our previous study (7). Briefly, all strains were recovered on yeast-peptone (YP) agar medium from freeze-dried stocks; these were cultivated in YP broth at 27°C for 1 day with agitation at 140 rpm. Then, 1-ml aliquots of each culture were used for genomic DNA extraction with a DNeasy minikit (Qiagen, Hilden, Germany). The DNA libraries were prepared from genomic DNA using an Ion Plus fragment library kit, with physical shearing and size selection (about 200 bp), and were sequenced using an Ion PGM sequencer with an Ion PGM Hi-Q View OT2 kit, an Ion PGM Hi-Q View sequencing kit, and an Ion 318 Chip kit v2 (all from Thermo Fisher Scientific, Inc., Waltham, MA, USA), according to the manufacturer’s instructions. The sequence reads were evaluated for quality (quality scores of <20) and adapter sequences were trimmed using CLC Genomics Workbench v12 (Qiagen). Using these reads, multiple contigs were assembled *de novo* using the same software with default parameters (mapping mode = create simple contig sequences [fast], automatic bubble size = yes, minimum contig length = 500, automatic word size = yes, performing scaffolding = yes, auto-detect paired distances = yes). Using the CLC Genomics Workbench program, we confirmed that the genome coverage of these contigs is sufficient for genome mapping and that the correctness of these contigs is maintained by removing suspected contamination sequences. The draft genomes were annotated using the NCBI Prokaryotic Genome Annotation Pipeline (PGAP) v4. 1 (8).

The G+C contents and genome sizes for these strains were found to be 58.5 to 58.7% and 4.2 to 6.1 Mbp, respectively (Table 1). PGAP identified 5,662 to 6,081 genes, including multiple rRNA and tRNA genes. Various polymorphisms were detected in the Pac_ICE1 regions of these strains except for MAFF 212115 and MAFF 212118, in which Pac_ICE1 could not be detected. Further investigations are needed to determine whether MAFF 212115 and MAFF 212118 possess Pac_ICE1. Other than Pac_ICE1, no ICEs were detected in the nine draft genomes sequenced in this study. This information will contribute to future studies on the genomics of biovar 3 worldwide.

### Data availability.

All sequences identified in this study have been deposited in GenBank (see Table 1 for accession numbers).
